# A bibliometric analysis of the top 50 cited studies related to acromioclavicular joint instability

**DOI:** 10.1016/j.jor.2024.06.037

**Published:** 2024-06-27

**Authors:** Conor J. Kilkenny, Fahad Farooq, Eoghan T. Hurley, Gordon R. Daly, Gavin P. Dowling, Sean P. Whelehan, Hannan Mullett

**Affiliations:** a Royal College of Surgeons in Ireland, Ireland; b SUNY Upstate Medical University, Syracuse NY, USA; c Duke University Medical Center, Erwin Rd, Durham, USA; d University of Limerick School of Medicine, Limerick, Ireland; e UPMC Sports Surgery Clinic Santry, Dublin, Ireland

**Keywords:** Acromioclavicular joint, Shoulder, Sports injury, Instability, Treatment outcomes

## Abstract

**Background:**

Acromioclavicular joint (ACJ) injury is a common orthopaedic condition accounting for over 40 % of all shoulder injuries. The purpose of this study is to assess the research trends and characteristics of the top 50 cited articles on ACJ instability.

**Methods:**

A systematic search was conducted in Web of Science to identify articles primarily related to ACJ injury or instability. Characteristics including citation number, country of origin, journal and institution of publication, impact factor, authorship, level of evidence, patient demographics, and study type were analyzed and recorded.

**Results:**

Research output on ACJ instability has been steadily increasing, with the top 50 cited studies predominantly presenting Level IV evidence. These studies primarily focused on treatment outcomes which included predominantly male patients and exhibited a large variation in citation counts. The American Journal of Sports Medicine was the most productive journal, and the USA was the most productive nation.

**Conclusion:**

There is an increasing number of publications in the ACJ instability literature, primarily concentrated in a few institutions and journals, and focusing mainly on treatment outcomes. A significant portion of these publications are of low scientific quality, and there is a notable lack of research on outcomes for females.

## 1 Introduction

Acromioclavicular joint (ACJ) injury is a common orthopedic condition accounting for over 40 % of all shoulder injuries.^1^ There is a varying degree of morbidity associated with ACJ injury, based primarily on disease severity. Mild disease (sprains of ligaments supporting the joint) often results in good outcomes, and severe disease (dislocations, tears) often leads to significant impairment of shoulder function, strength, and lifestyle.^1^ ACJ instability frequently results from trauma or other sport-related activity, with lesser incidence due to osteoarthritis.^2^ Young male athletes who participate in sports have a five-time higher incidence of ACJ injury than their female competitors.^3^ Both acute and chronic injury to the ACJ has the potential to have severe consequences on shoulder function, therefore it is crucial that appropriate and prompt treatment is provided to preserve functional status.^4^ Many surgical approaches have been trialed and utilized to treat patients suffering from chronic and symptomatic ACJ instability.^5^ This involves open and arthroscopy-assisted procedures, anatomical and non-anatomical techniques as well as biological and synthetic grafts.^6^ These treatment options have been widely studied and evolved over the years, with research ranging from basic science investigations and case reports to clinical trials.

The quantity of citations an article receives is often used as a measure of its scientific influence.^7^^,^^8^ This method of classifying articles based on citation count is known as a bibliometric analysis. Bibliometric analyses are now a recognised means of evaluating and visually presenting research trends and noteworthy contributions of a particular topic. These analyses have been performed for other common orthopedic topics such as joint arthroplasty (particularly of the knee, hip, and shoulder) and surgical techniques within orthopaedics.^9^, ^10^, ^11^ However, no prior bibliometric analysis of ACJ instability has been conducted.

The purpose of this study is to assess the research trends and characteristics of the top 50 cited articles on acromioclavicular joint instability. It was hypothesized that the majority of published work on ACJ instability would have occurred in the last decade and involved a limited number of institutions and journals.

## 2 Methods

### 2.1 Study selection & search strategy

Two independent reviewers performed a search of The Web of Science Core Collection databases (Clarivate Analytics, Boston, MA, USA) in April 2022. The search terms included “Acromioclavicular joint dislocation” OR “Acromioclavicular joint instability” OR “Acromioclavicular joint disruption” OR “Acromioclavicular joint injury”. No time limit was given to publication date.

### 2.2 Eligibility criteria

The inclusion criteria were the following: (1) Original research with primary focus on ACJ injury or instability, (2) published in a peer-reviewed journal, (3) published in English, and (4) full text of study available. The exclusion criteria were the following: (1) review/meta-analysis studies, (2) biomechanical studies, (3) anatomical/cadaveric studies, and (4) abstract only available. For the purposes of this analysis, the term “injury” includes damage or instability related primarily to the bony structures, ligaments, and musculature surrounding the ACJ, specifically sprains, stretching, tearing, fracture, displacement, or separation of the joint.

Articles that met these criteria were screened by two independent reviewers and cross-checked for consistency. The top 50 most cited articles were extracted into Microsoft Excel (Microsoft Corporation, Redmond, WA) for analysis.

### 2.3 Study characteristics

The following characteristics were analyzed and recorded from each study: citation number, country of origin, journal and institution of publication, impact factor of journal, author, focus of study, level of evidence, patient demographics, type of study, patient number and mean time to follow-up. To reveal the research focus, major topics of each article were identified through a review of article objectives and keywords. The level of evidence was determined based on the criteria by Wright et al.^12^

### 2.4 Statistical analysis

The VOSviewer 1.6.16 software (Leiden University, Netherlands) was employed to conduct a visual study of the top 50 cited articles. This study aimed to interpret the data and identify connections among them. To evaluate the contributions of authors and institutions, a full-counting bibliographic coupling analysis was conducted. Additionally, a full-counting co-occurrence analysis was employed to identify research hotspots based on the keywords. Finally, co-citation analysis was performed to establish relationships among the authors cited in the articles.

## 3 Results

### 3.1 Literature search

**Fig. 1:**
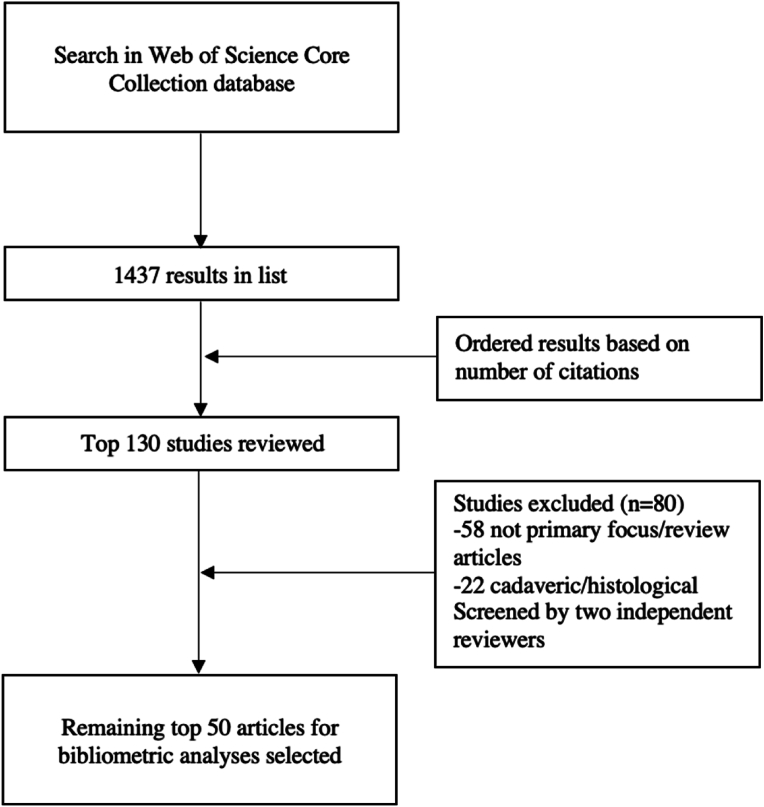
Flowchart representing search screening and selection criteria.

The search strategy yielded 1437 studies which were sorted through and filtered based on criteria for selection. After sorting by number of citations, the top 130 studies were evaluated and 80 studies were excluded. The remaining 50 full texts were included in this review (Fig. 1).

### 3.2 Study characteristics & patient demographics

The number of citations per study varied widely, with the most cited study having 349 citations, and the least cited having 52. Nearly one-quarter of all papers had over 100 citations. Treatment outcomes were a major focus of many studies examined, with the fewest focusing on epidemiology of ACJ instability. Patient gender was recorded in 32 studies. 870 patients were male, while 99 patients were female (Table 1).

**Table 1:** Summary of bibliometric parameters of the top 50 cited studies.

Characteristic	Count
Number of citations total (range)	4866 (52–349)
No. of studies with >100 citations (% of total)	12 (24)
Patient	
Total Number (range)	1975 (1–318)
Mean Age	37.2
Sex	
Male (No. of studies)	870 (32)
Female (No. of studies)	99 (32)
Focus, No. of studies (% of total)	
Diagnostic	2 (4)
Treatment outcomes^x^	36 (72)
Surgical complications	2 (4)
Associated injuries	3 (6)
Surgical technique	6 (12)
Epidemiological	1 (2)
Time to follow-up	
No. of studies examined	33
Mean, months (range)	40.5 (9.7–150)

x Contained studies considering conservative management, surgical management, and treatment comparison studies.

**Fig. 2:**
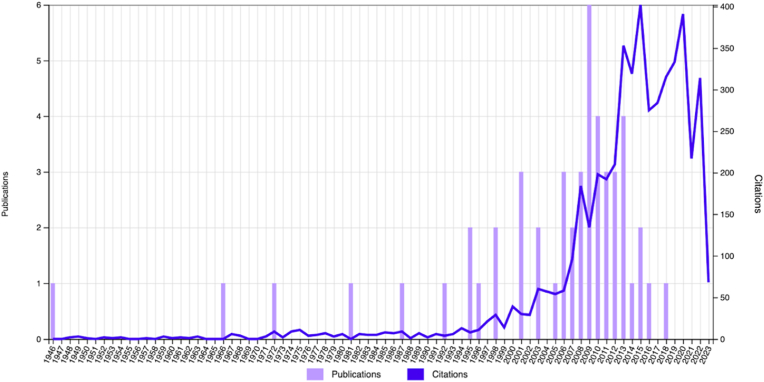
Publication trends over time related to ACJ injury.

**Fig. 3:**
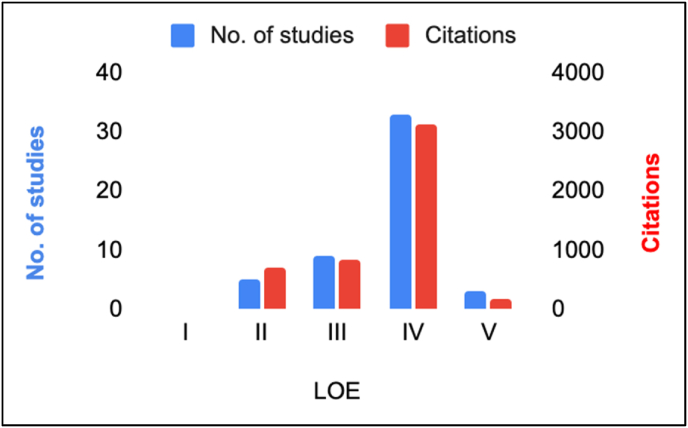
Trends of ACJ Published Studies and their Levels of Evidence (LOE).

**Fig. 4:**
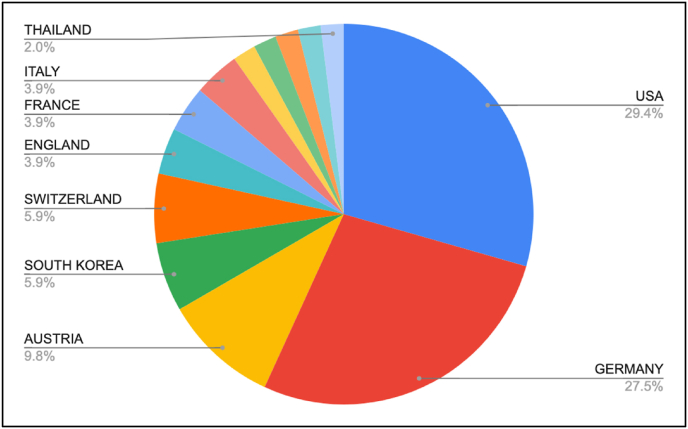
Percentage of Top 50 Most Cited Articles from Each Country.^1^ Three articles did not include the country of publication and were excluded from this analysis.

There has been an upward trajectory in the number of published papers from 1946 onwards, with a decline observed since 2019. The two most cited studies originated prior to the 2000s (1972 and 1998, respectively) (Fig. 2). A large percentage (64.5 %) of the top 50 studies were of Level IV evidence (Fig. 3). The author AB Imhoff from the Technical University of Munich had the highest number of citations (Table 2). This institution also had the highest number of overall publications (Table 3). The most productive journal was the American Journal of Sports Medicine, with 21 studies and with the highest impact factor (Table 4). Thirteen countries contributed to the top 50 cited articles on ACJ instability. The USA produced the largest number of papers, with 15 of the 50 studies (29 %). Germany followed with 14 articles (28 %) (Fig. 4).

**Table 2:** The top 5 most frequently cited and published authors.

Author	Institution	No. of articles	No. of citations
Imhoff, AB	Technical University of Munich	5	508
Salzmann, GM	Technical University of Munich	4	431
Tauber, M	ATOS Clinic Munich	4	413
Scheibel, M	Charite Universitatsmedizin Berlin	3	370
Resch, H	Paracelsus Medical University	3	348

**Table 3:** The top 5 institutions with frequently cited articles.

Institution	Country	No. of articles
Technical University of Munich	Germany	5
Charite Universitatsmedizin Berlin	Germany	3
Free University of Berlin	Germany	3
Humboldt University of Berlin	Germany	3
Paracelus Private Medical University	Austria	3

**Table 4:** Top 10 most productive journals with impact factor.

Journal	No. of studies	Impact Factor^a^
American Journal of Sports Medicine	21	7.01
Arthroscopy The Journal of Arthroscopic and related surgery	7	4.433
Journal of Shoulder and Elbow Surgery	5	3.883
Knee Surgery Sports Traumatology Arthroscopy	5	4.407
Archives of orthopedic and trauma surgery	3	3.404
Journal and bone and joint surgery (American volume)	3	6.588
Clinical orthopedics and related research	2	4.837
ACTA Orthopaedica Belgica	1	0.365
European Journal of Medical Research	1	4.981
Journal of Bone and Joint Surgery	1	6.588

a From 2023–2023.

A coupling analysis of institutions and authors showed a total of seven institutions that met a threshold of a minimum of two articles, with the highest Link Strength (LS) institution being Technical University Munich, with four publications, 397 co-citations, and LS = 209. Second and third were University Hospital Salzburg (citations = 208, LS = 136) and Tripler Army Medical Center (citations = 121, LS = 109), with two articles each.

There were 23 articles that met a minimum threshold of two articles for author coupling analysis. The highest link strength author was Imhoff AB, with five publications and 508 citations (LS = 1562). Second and third were Salzmann GM (LS = 1236) and Walz L (LS = 1236) with 3 publications each.

A total of 168 keywords were analyzed for frequency of occurrence. The top five most frequently used keywords were “shoulder” (n = 18, LS = 164), “reconstruction” (n = 15, LS = 145), “injuries” (n = 13, LS = 135), “dislocation” (n = 13, LS = 125), and “acromioclavicular joint” (n = 12, LS = 120).

**Fig. 5:**
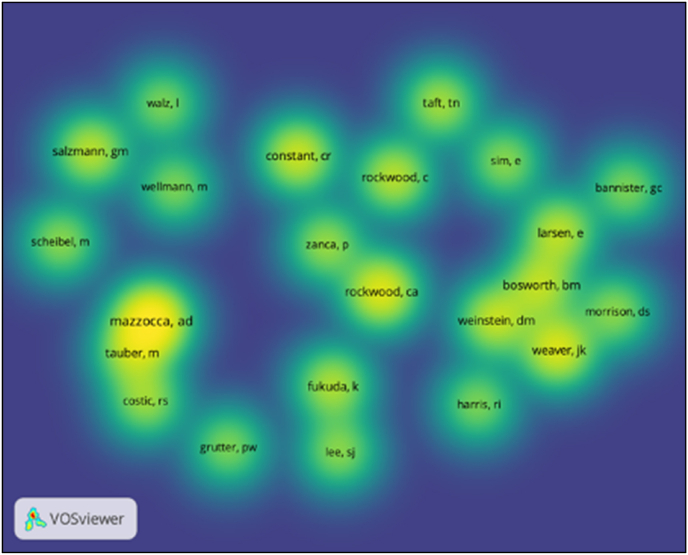
Co-citation analysis of authors.

Using a minimum of 10 citations, 23 authors were included in a co-citation analysis (Fig. 5). The top five authors according to link strength were Mazzocca AD (citations = 28, LS = 223), Constant CR (citations = 17, LS = 140), Rockwood CA (citations = 20, LS = 139), Weaver JK (citations = 19, LS = 138), Salzmann GM (n = 15, LS = 129). Among the top three most co-cited authors, two were from the USA (Mazzocca AD, Rockwood CA) and one from the UK (Constant CR).

## 4 Discussion

The most important finding of this study is an increasing number of publications in the ACJ instability literature, primarily concentrated in a few institutions and journals, and focusing mainly on treatment outcomes. A significant portion of these publications are of low scientific quality, and there is a notable lack of research on outcomes for females. Despite an increasing interest in this research topic over time, only about one-tenth of total patients found in these top 50 studies were female.

A number of recent papers have found an increased incidence of ACJ injury among young males, suggesting risk factors for injury to the ACJ include male gender and younger age group.^13^^,^^14^ The increased incidence of ACJ instability in males likely contributes to their higher representation in research studies. The most common mechanism of injury of the ACJ is a result of direct trauma to the lateral aspect of the shoulder,^1^ and most of these injuries occur during contact sports like rugby, wresting, and hockey.^13^ Females are thought to have increased susceptibility to multidirectional shoulder instability due to their association with increased joint laxity.^15^, ^16^, ^17^ Although sexual dimorphisms in many orthopedic pathologies have been documented in the literature,^15^ there is limited research on if gender differences impact management of ACJ injury. Previous studies within orthopaedics have shown that outcomes differ based on sex.^18^ They demonstrated that female patients required an increased hospital length of stay, increased opioid usage, and higher rates of discharge to long term care facilities when compared to their male counterparts.^18^, ^19^, ^20^, ^21^, ^22^ Despite this, Matzkin et al. found that many orthopedic surgeons do not routinely consider the sex of a patient in evaluation and treatment plans for patients with musculoskeletal disorders.^23^ Overall, there exists a need to conduct more inclusive studies which focus on outcomes and treatment for female patients with ACJ instability.

ACJ instability remains an ongoing challenge for shoulder surgeons, with no agreed standard of care.^24^^,^^25^ There is still a scarcity of high-level research in this field, with this analysis demonstrating that the majority of studies are classified as Level IV evidence. This is similar to bibliometric analyses conducted in other orthopedic specialties.^9^^,^^10^^,^^26^ The keyword co-occurrence analysis emphasizes the significance of optimizing surgical outcomes, addressing severe cases, and exploring advancements in surgical techniques and implants. Currently, there is no gold-standard treatment of ACJ separation.^27^ Therefore the need for evidence-based guidelines and standardized protocols in the field are essential.^28^

Citation counts appear to vary greatly within orthopaedics depending on the subspecialty. Moore et al. conducted a bibliometric analysis on shoulder arthroscopy which found the most cited study was cited 1134 times,^11^ while a similar study performed by Constantinescu et al. on hip arthroscopy showed an even greater citation number with 4702 citations.^29^ The top cited study in this ACJ analysis had only 349 citations. This suggests further research is needed to strengthen this area of ACJ instability. It also supports Haimowitz et al.‘s findings that orthopedic subspecialties have different levels of research output.^30^

The ACJ instability literature exhibits a skewed citation distribution, wherein a select few papers have garnered a considerably higher number of citations compared to the majority. This suggests the presence of influential works that have significantly impacted the field.^23^ Notably, older studies have contributed to this impact, but their relevance stems from introducing novel findings or techniques rather than solely due to their longevity. For instance, the top-cited paper by Weaver and Dunn introduced the pioneering “Weaver-Dunn” technique, which, until recently, was a widely employed technique for treatment of ACJ separation.^28^^,^^31^, ^32^, ^33^ Many of the top co-cited studies have made substantial contributions to the field of orthopedics. For instance, Rockwood devised the widely used Rockwood classification system for ACJ fractures.^34^ The co-citation analysis also revealed instances where authors within the same network collaborated and published multiple papers together, demonstrating a strong collaborative trend within the field.

The American Journal of Sports Medicine has been prominent for disseminating research on ACJ instability. This is similar to other bibliometric analyses within orthopaedics likely reflecting its reputation within orthopedics and high impact factor.^11^^,^^35^^,^^36^ This is also consistent with previous research showing citation frequency is influenced by the impact factor of the journal in which it is published.^37^ As in many other orthopaedic bibliometric analyses, the USA produced the greatest number of studies, and this pattern is expected given the resources the USA has available.^38^^,^^39^

### 4.1 Limitations

There are some important limitations of this study. First, the focus on the Web of Science Core Collection databases may not capture all relevant articles on ACJ injury. Given that this analysis revealed a decline in ACJ instability research output since 2019, this may reflect a reduction in overall orthopedic research related to the COVID-19 pandemic or the existence of papers published beyond the scope of this bibliometric analysis.^40^ Second, the exclusion of non-English articles may also lead to language and publication bias. Finally, citation count as a measure of impact may not fully capture the quality or clinical significance of a study. Given the nature of this analysis, recent papers in high impact journals, with notable value to the literature may be excluded given their low citation count.

## 5 Conclusion

There is an increasing number of publications in the ACJ instability literature, primarily concentrated in a few institutions and journals, and focusing mainly on treatment outcomes. A significant portion of these publications are of low scientific quality, and there is a notable lack of research on outcomes for females.

## Conflict of interest

None.

## Funding/sponsorship

This research did not receive any specific grant from funding agencies in the public, commercial or not-for-profit sectors.

## Informed consent (Patient/Guardian), mandatory only for case reports/clinical images

N/A.

## Institutional ethical committee approval (for all human studies)

N/A.

## Informed consent

Not applicable.

## Ethical statement

1) This material is the authors' own original work, which has not been previously published elsewhere.
2) The paper is not currently being considered for publication elsewhere.
3) The paper reflects the authors' own research and analysis in a truthful and complete manner.
4) The paper properly credits the meaningful contributions of co-authors and co-researchers.
5) The results are appropriately placed in the context of prior and existing research.
6) All sources used are properly disclosed (correct citation). Literally copying of text must be indicated as such by using quotation marks and giving proper reference.
7) All authors have been personally and actively involved in substantial work leading to the paper, and will take public responsibility for its content.

## CRediT authorship contribution statement

**Conor J. Kilkenny:** Investigation, Writing – original draft, Visualization. **Fahad Farooq:** Investigation, Methodology, Writing – review & editing. **Eoghan T. Hurley:** Conceptualization, Writing – review & editing. **Gordon R. Daly:** Methodology, Formal analysis, Investigation, Writing – review & editing. **Gavin P. Dowling:** Investigation, Formal analysis, Writing – review & editing. **Sean P. Whelehan:** Formal analysis, Investigation, Writing – review & editing. **Hannan Mullett:** Conceptualization, Writing – review & editing, Supervision.
